# On-the-Pack Voluntary Well-Being Messaging for Milks Targeting Chinese Older Adults: A Content Analysis

**DOI:** 10.3390/foods11152212

**Published:** 2022-07-25

**Authors:** Ao Chen, Saleh Moradi, Joanne Hort

**Affiliations:** 1 Food Experience and Sensory Testing (Feast) Lab, Massey University, Palmerston North 4442, New Zealand; a.chen2@massey.ac.nz (A.C.); sal.moradi@fonterra.com (S.M.); 2 Riddet Institute, Massey University, Palmerston North 4442, New Zealand; 3 Fonterra Research and Development Centre, Fonterra Co-Operative Group Limited, Private Bag 11029, Palmerston North 4410, New Zealand

**Keywords:** dairy, China, well-being message, consumer insights, food safety national standards

## Abstract

China is experiencing severe population aging. Given that milks targeting older adults are one of the most popular foods designed for Chinese older adults (COA), this study investigated on-the-pack (OTP) voluntary well-being messaging (VWM, ways of communicating a product’s broad well-being benefits through information on food content or statements linked to favourable components, functions, or well-being outcomes) for milk targeting COA. Over 200 products identified from two sources (JD.com and Mintel’s global new products database), were analysed for type, content, and VWM frequency for different brand origins and milk sources of various animal species, nutrition claim regulation compliance and alignment with nutrition facts. The results suggested: (1) different brand origins (domestic vs. international) and milk source (cow vs. goat) highlighted different well-being aspects of products, (2) three products failed to comply with government regulations made for nutrition labelling of pre-packed foods (GB 28050-2011), but (3) excepting fat, all ‘contains’ claims and most ‘high’ claims did not reflect significantly greater levels of nutrients, compared to products with no claims. The findings create a comprehensive picture of OTP VWM for milks targeting older adults in China, providing useful information for consumer, domestic, and international dairy industries, and policymakers.

## 1. Introduction

Milk, the primary source of nutrition for young mammals, is also a key nutritional component of the diet in many cultures across the globe, obtained mainly, but not exclusively, from goats and cows [1]. Despite recent disputes, mounting evidence from various research disciplines continues to support milk’s unique nutritional value contribution [1,2,3] and links between its consumption and both physical and psychological benefits [1,4,5,6]. Although there is variation across species, milk, provides a source of proteins, carbohydrate, lipids, minerals, especially calcium and phosphorous, and vitamins [1,2,7]. Its consumption is also associated with reduced incidence of colorectal, bladder, and gastric cancer, reduced risk of obesity, diabetes, cardiovascular disease, frailty, and sarcopenia, and improved metabolism, bone health, gut function, and cognitive performance [1,4,5,6]. Milk products are also regularly fortified with nutrients including vitamin D, calcium, iron, magnesium, zinc, phytosterol, to add further to their nutritional value and well-being functions whilst, importantly, retaining their sensory and physiochemical properties [8,9,10,11,12].

These qualities of milk, when considered alongside common age-related health issues such as musculoskeletal disorders, sarcopenia, chewing and swallowing difficulties and gastrointestinal tract functional disorders [13,14,15,16], make milk an ideal candidate to deliver vital nutritional and functional components needed for healthy-ageing, which was defined as “leading a healthy, active, social, and independent life in later years, through maintaining vitality and good quality of life for as long as possible” [17]. This is echoed in consumer studies. A recent study of lower income older adults in the US found that primary reasons for milk consumption included that it was “good for bones/osteoporosis”, “good for health”, “a source of calcium”, and “what the doctor recommended” [18]. Among Scottish older adults, milk was consumed by most individuals (97%) and, therefore, was considered an important staple food for this age group [19]. In China, milk powder is one of the most popular food products designed for older adults, along with pastry and soup [20,21]. Mintel’s global new product database (GNPD) recorded nearly one in every three (100 out of 344) food products targeting older adults launched globally as being a liquid and powdered milk targeting older adults in mainland China (hereafter China) in the past five years (i.e., 2016–2020). 

In a crowded market, industry strives for production, marketing and retailing strategies that create meaningful points of differentiation with competitors. Voluntary well-being messaging (VWM), that is, voluntary ways to communicate broad well-being benefits of specific products to the consumer through information concerning food content, or statements that link the product with favourable components, functions or well-being outcomes, is a marketing strategy commonly prescribed for product success in competitive markets. Specifically, on-the-pack (OTP) VWM creates competitive advantage through informing consumers of the purported health and nutritional value, reliability and food safety of the product at the point of choice, which has been confirmed by using eye-tracking technology in consumer studies [22,23]. For milk, OTP VWM has been shown to influence milk consumption behaviour, purchasing behaviour, and willingness to pay [24,25].

Notably, VWM can be communicated indirectly and via seemingly unrelated packaging design features. Examples include images of happy and healthy individuals, schematic drawings of a body organ, informal diet and lifestyle advice, or vision and mission statements of the manufacturer. This type of VWM becomes more prominent, and potentially more effective, considering that reading, analysing, and interpreting more technical nutrition information tables, often in small font sizes on the back-of-pack (BOP) can be a challenge for the average consumer. More importantly, prior research suggests that ‘soft’ VWM, information that consumers loosely associate with health without there being a proven effect, can, in certain contexts, be more powerful than ‘scientific’ health claims in influencing positive health inferences about the product [26]. Among less educated consumers, for instance, front-of-pack (FOP) VWM presenting a product’s key nutrients in a simplified format, played a more significant role than nutrition information tables in choosing healthy foods [27]. A study, surveying a large sample of consumers in 10 cities across five provinces in China, found that consumers used simplified FOP VWM significantly more frequently than detailed BOP nutrition information at point-of-purchase [28].

Whilst milk well-being messaging (WM) has been subject to research [29], messaging targeting older adults, and messaging in China’s market have both been overlooked. Considering the benefits that studying VWM for specific products targeting a specific segment in a particular market can offer for various stakeholders (consumer, industry, policy makers, and researchers) this gap warrants abridging. This study intends to introduce VWM as an umbrella term to capture various instances of voluntary ways to communicate the broad well-being benefits of products to the consumer regardless of type, format, origin, etc. This necessitates a content analysis of VWM appearing OTP of pre-packaged milk of any species that are claiming suitability for COA. 

As a key medium for informing consumers about food products [30,31], general and OTP and voluntary WM is often subject to strict local and international regulations. OTP use of mandatory and voluntary WM for food products is highly regulated across various legislation areas, such as the EU, the US and Japan [32]. In China, Food Safety National Standards for the labelling of pre-packed foods (GB 7718-2011) and Food Safety National Standards for nutrition labelling of pre-packed foods (GB 28050-2011) were implemented in 2012 and 2013, respectively, to standardise practice and improve the integrity of both voluntary and mandatory OTP WM, including but not limited to nutrition and nutrient function claims [33,34]. Whilst implementation of the Food Safety National Standards has led to enhanced use and standardisation of OTP WM for food products in China [35,36], the existence of national regulations, even in countries with a longer tradition of enforcing them, does not guarantee compliance. Prior research in Australian [37], Brazilian [38], and EU [39] markets has revealed various levels and types of OTP WM non-compliance. Whether milks targeting COA comply with current national regulations on VWM is currently unknown. 

Whilst regulation compliance is a valid research avenue, it does not ensure optimal communication of well-being information to the consumer. Regulatory frameworks are often successful in ensuring well-being message compliance, but implicit, technical, inaccurate, misaligned, and sometimes conflicting messages appear on packs, and can misinform, misguide or, at best, confuse consumers [40,41]. Prior research suggested that the average Chinese consumer, whilst showing higher purchase intent for products featuring nutrition claims, neither possesses the nutrition knowledge vital for clear interpretation of claimed information nor pays enough attention to nutrition information tables as a secondary source of data [42]. Therefore, it is important to test whether nutrition facts on milks for COA are in line with nutrition claims. 

Accordingly, the aims of this study were to (1) provide a comprehensive survey of pre-packaged milks that are (explicitly or implicitly) claiming suitability for older adults in China’s market; (2) perform a content analysis on those milks by systematically extracting all textual and graphical OTP VWM on milks targeting COA, whilst concurrently, creating a general framework for OTP VWM; (3) assess the compliance of OTP nutrition claims with national regulations; and (4) investigate the alignment of OTP nutrition claims with nutrition facts.

## 2. Materials and Methods

### 2.1. Identification of Products

Milks targeting COA commercially available in the market from 1 March to 10 June 2021 were included in this study. COA (in Chinese: 中老年) were defined as people residing in China aged 40 and older, the lower age limit claimed on the packs of eligible products. Products that clearly mentioned COA or claimed suitability for this age group on the pack were included. Additionally, products whose suitability for COA could be clearly implied from either the product name or images of older adults on the pack were also considered. Milk had to be the main ingredient of the product (≥80%), so plant-based alternatives and combined milk/plant products were excluded. Additionally, dietary supplements were not included since they are not classified as food.

Two data sources were used to identify products meeting the above criteria: JD.com and Mintel GNPD. JD.com is one of the two largest business-to-consumer online retailers in China. In the financial year of 2020, JD.com’s annual active customer accounts reached 471.9 million with the annual net revenues of 114.3 billion USD [43]. The key words: *中老年* and *奶* (in English: *older adults* and *milk*) were searched amongst product titles on JD.com. Mintel GNPD is a global online database of packaged food and beverage, used as a reliable and comprehensive source of products available in various markets around the world, with over 40 thousand new products added monthly and over 5 million existing records [44]. The key words: *middle*, *elderly*, *older* and *senior* were searched on Mintel GNPD in the field of “product name” and “product description”, under the region of “China” and the category of “food-dairy” excluding “plant-based alternative”. 

Search results were further refined for all forementioned criteria manually. Eligible products in their latest packaging were selected. Identical products in different sizes, with different packaging materials and from different stores on JD.com were considered duplicates and recorded only once.

### 2.2. Information Extraction

#### 2.2.1. Product Information

Detailed product information was extracted from JD.com and Mintel GNPD website, including food category (i.e., liquid milk or milk powder), brand origin (i.e., international or domestic), milk source (i.e., cow, goat, sheep, etc.), nutrition facts and package images. Brand origin was identified according to OTP manufacturer information. Products with brands originating in China were classified as domestic products and the rest as international products. Food category and milk source were identified from the product name and OTP ingredients list. Nutrition facts were sourced from the OTP nutrition information table. Official and unofficial detailed package images were obtained from Mintel GNPD or JD.com product descriptions and customer reviews sections. 

#### 2.2.2. Voluntary Well-Being Messaging Elements

**Figure 1:**
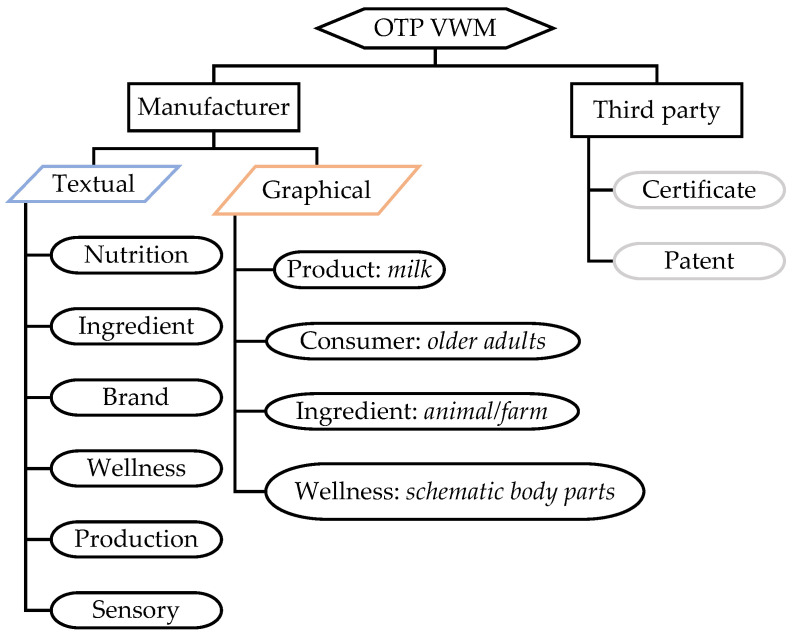
Classification of on-the-pack (OTP) voluntary well-being messaging (VWM) on milks targeting Chinese older adults.

**Figure 2:**
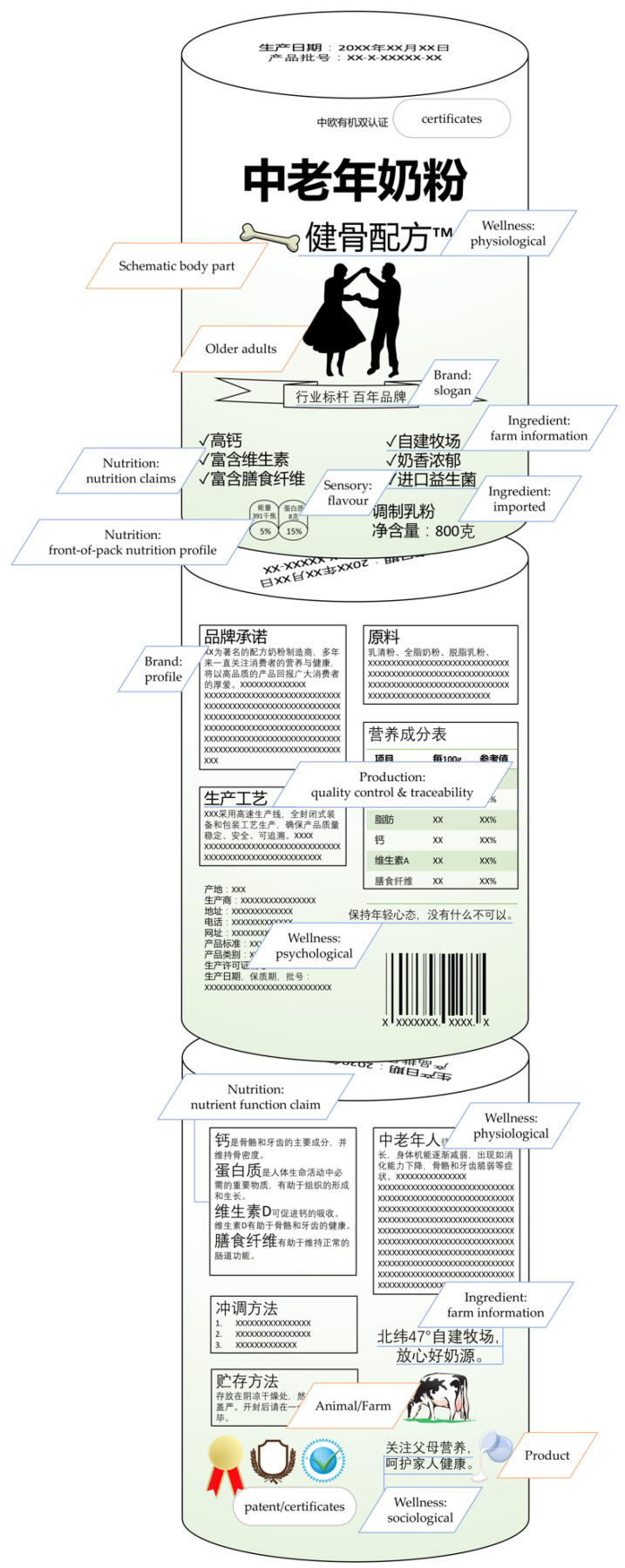
On-the-pack voluntary well-being messaging: textual (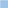), graphical (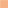) from manufacturers and certificates and patent from the third parties (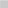) as extracted from an example milk targeting Chinese older adults.

As this research aimed exclusively at analysing VWM, mandatory instances of WM, for example, ingredients list, nutrition information tables, manufacturer information, net weight, production date and shelf-life were not considered. For the content analysis, the incidence of all OTP VWM was taken from product packaging images. A framework was developed to indicate how the content was sourced and classified (Figure 1). VWM provided by manufacturers was classified as either textual or graphical. Textual VWM included six aspects: nutrition, ingredient, brand, wellness, production and sensory. For international brands, textual VWM was recorded only if it appeared in Chinese as information provided in other languages was not deemed readily interpretable to average Chinese consumers. Exceptions to this rule were symbols for elements and abbreviations of nutrients commonly used in China, such as Ca for calcium and VD for vitamin D. Graphical WM included images of milk (the product), older adult (the consumer), animal/farm (the ingredients) and schematic body part (wellness). Third party VWM included certificates and patents, which were either textual, graphical or both (e.g., symbols and logos), such as International Organization for Standardization (ISO) certification, organic food certificate, New Zealand made triangle, Halal logo and recycle symbols. Detailed classifications and working definitions developed for the textual VWM aspects are listed in Table 1 with illustrative examples. Furthermore, an example product is shown in Figure 2, with instances of types of VWM indicated.

### 2.3. Compliance and Alignment of Nutrition Claims

#### 2.3.1. Compliance with the Food Safety National Standards

According to Food Safety National Standards for nutrition labelling of pre-packed foods (GB 28050-2011) [34], nutrition claims are categorised as content claims and comparison claims with criteria (Table 2). Food Safety National Standards are enforced by law and administrative regulations [28]. 

#### 2.3.2. Alignment with Nutrition Facts

Alignment between level of nutrition claim and nutrition facts were further investigated by comparing the values provided in OTP nutrition information tables amongst milk powders making different nutrition claims (liquid milks were not considered as they were not comparable to milk powders in terms of nutrient concentrations). Given that most nutrient concentrations were not normally distributed, nonparametric statistics were employed: Mann–Whitney *U* test for two levels of nutrition claims and Kruskal–Wallis *H* test for three levels of nutrition claims [45]. Dunn’s post hoc tests were carried out with adjustments using Bonferroni correction [46]. Effect sizes were estimated using eta squared and epsilon squared, respectively [47]. A minimum sample size of five per nutrition claim level was imposed for statistical analysis [48]. Claims made on minerals *per se* and vitamins *per se* were treated as if the claim has been made on all individual minerals and vitamins for which concentrations were presented in the nutrition information table. Statistical analyses were performed using SPSS (IBM, version 25.0) (α = 0.05). 

## 3. Results

### 3.1. Products Summary

Summarised in Table 3, a total of 207 eligible products were identified: 130 products were exclusive to JD.com, 12 to Mintel GNPD, and 65 were identified from both. The majority were milk powders (99%), only three were liquid milks. There were 175 domestic products and 32 international products from New Zealand (10), Switzerland (7), Australia (6), the Netherlands (4), Taiwan (3), France (1), and the United States (1). Cow and goat were the major milk sources, representing 59% and 36% of all products, respectively. Yak, camel and sheep milks represented the remaining 5% of products.

### 3.2. Voluntarty Well-Being Messaging

**Figure 3:**
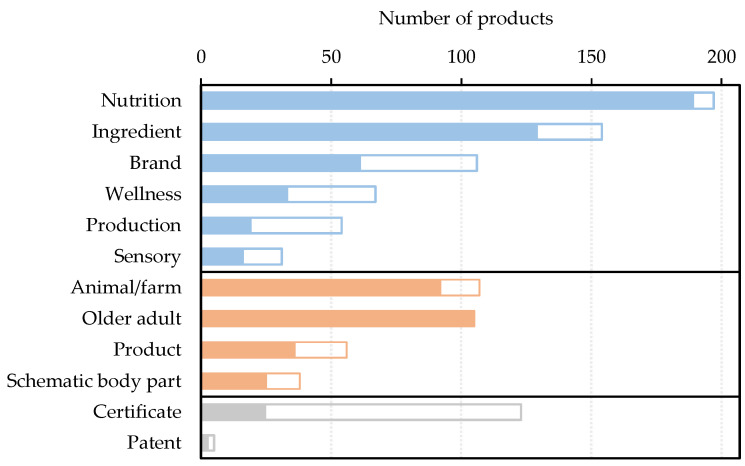
Voluntary well-being messaging on milks targeting Chinese older adults: textual (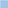), graphical (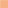) from manufacturers and certificates and patent from the third parties (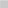) on the front-of-pack (solid bars) or not (open bars).

In line with the classification framework devised in this study (Figure 1), manufacturer originated VWM (either textual, graphical or both) was employed by all products, whilst third party originated VWM only appeared on 62% of all products. Figure 3 demonstrates the number of products displaying VWM in details. Amongst textual well-being messages, nutrition was the most popular aspect, appearing on 95% of all products, followed by ingredient (74%), brand (51%), wellness (32%), production (26%) and sensory (15%). Images of animals/farms (53%) and older adults (52%) were found on more than half of all products. In this case, 96%, 84% and 58% of nutrition-, ingredient- and brand-related textual VWM were found on FOP, respectively, and the other aspects of textural VWM were more frequently found on the other sides only. Most graphical well-being messages were on FOP, whilst the majority of third party generated VWM were not.

#### 3.2.1. Textual VWM from Manufacturers

OTP textual VWM on milks targeting COA are listed in Table 4 with the percentage of products by total, brand origin and milk sources. 

**Figure 4:**
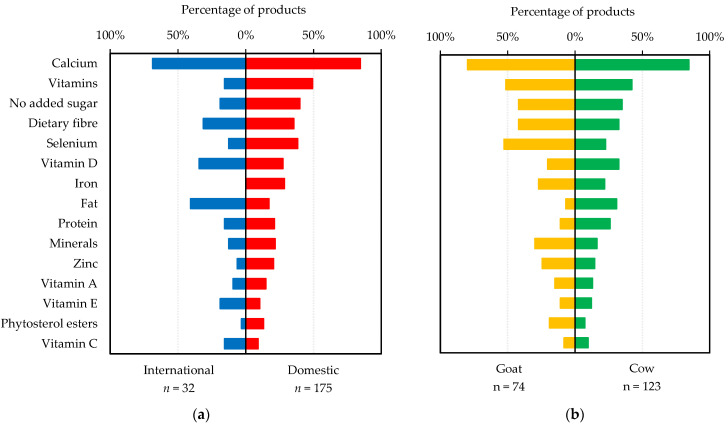
Percentage of milks targeting Chinese older adults made nutrition claims on specific nutrients that appeared on at least 5% of all products: (**a**) international brand vs. domestic brand; (**b**) goat milk vs. cow milk.

**Nutrition:** Nutrition-related messages consisted of nutrition claims, nutrient function claims and FOP nutrient profiling. A larger proportion of domestic products made nutrition claims than international products (98% vs. 78%). Proportionally, more domestic products made claims for *vitamins* per se (49% vs. 16%), whilst more international products made claims on specific vitamins (Figure 4a). Considerable differences existed between domestic and international products regarding minerals (Figure 4a), with no international product making a claim concerning iron. International products made more claims about fat content (low in fat or reduced fat) compared to domestic ones (38% vs. 17%), whilst ‘no added sugar’ claims were more prevalent amongst domestic than international products (40% vs. 19%). Most both cow (95%) and goat (97%) milks made nutrition claims. However, vitamins, dietary fibre, no added sugar, selenium, zinc, minerals and phytosterol esters were claimed more frequently for goat than for cow milks (Figure 4b). Calcium was the most mentioned nutrient in nutrition claims, irrespective of brand origin or milk source (Figure 4a,b).

**Figure 5:**
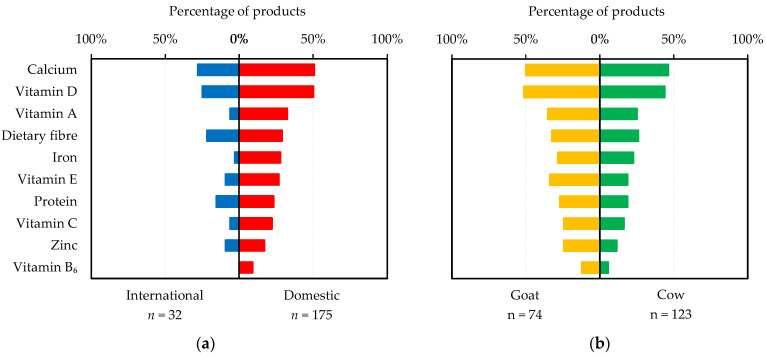
Percentage of milks targeting Chinese older adults made specific nutrient function claims that appeared on at least 5% of all products: (**a**) international brands vs. domestic brands; (**b**) goat milks vs. cow milks.

Nutrient function claims were made more often by domestic than international products (61% vs. 41%, Figure 5a). Proportionally, cow and goat milks were similar in terms of making nutrient function claims (58% vs. 59%, Table 3). However, a greater proportion of goat milks made nutrients function claims for each individual nutrient (Figure 5b), suggesting, in general, greater numbers of nutrients were mentioned in nutrient function claims made by goat than cow milks. Calcium and vitamin D were the most popular nutrients when making function claims regardless of brand origin or milk source (Figure 5a,b). 

FOP nutrient profiling was only found on eight products, and all were international brand cow milks (Table 3).

**Ingredient:** Expectedly, more international products claimed use of imported ingredients than domestic ones (78% vs. 15%). There were only three international products made from non-cow milk sources, hence the proportions of goat and other milk sources claiming imported ingredients were lower than cow milk (34% vs. 11%).

Only one international product provided messages concerning the farm, and as for different milk sources, farm messaging was more prevalent amongst goat and other animals than cow milks (43% and 50% vs. 27%). Similarly, a greater proportion of domestic products (42% vs. 31%) and goat milks (47% vs. 38% of cow and 20% of other milks) mentioned other specified ingredients than their counterparts. 

**Brand:** Similar proportions of domestic and international products provided information about their brand (51% vs. 50%) in general, but fewer international products stated their brand and company profile on the pack. Proportionally, goat milks provided less brand-related VWM than other milk sources, especially, in terms of having a slogan (Table 4).

**Wellness:** Although there were similar proportions of domestic and international products providing wellness-related messages (32% vs. 34%), they tended to be focused on sociological and psychological wellness, respectively. Notably, 11 domestic products exclusively cited seven pieces of sociological wellness messages about the family (Table 5). Compared with other milk sources, cow milks provided more wellness-related messages across all three physiological, psychological and sociological aspects. 

**Production:** No international products provided production-related messages, whilst domestic products frequently mentioned quality control (11%), traceability (4%) and other specific production and processing methods (23%). Similar proportions of goat and cow milks provided production-related messages, and as expected, mutton-flavour removal techniques were exclusively cited on goat milks (14%). 

**Sensory:** 15% of products displayed sensory-related messages and most of these described flavour credentials, which may reflect that manufacturers do not consider taste to be a key reason for COA to consume milk. This was not affected by brand origin (domestic: 15%, international: 13%), but an emphasis on mutton-flavour removal meant goat milks had more added opportunity for sensory-related messaging (20% vs. 13%).

#### 3.2.2. Graphical VWM from Manufacturers

Table 6 summarises the percentages and number of products displaying graphical VWM and their nature. 

**Images of older adult:** Images of older adults appeared more often on domestic products and cow milks than their counterparts. Photographs were more popular on cow milks, whilst cartoons were more frequently found on goat milks. Normally, the image of older adults was of two people (one male and one female). Where only one adult was shown, it was more likely to be a male on domestic products and a female on international products. Images of older adults with southeast Asian and European facial features appeared more often on domestic (35% vs. 13%) and international products (25% vs. 7%), respectively.

**Schematic body part:** Schematic body parts were more popular amongst international products (31% vs. 16%) and cow milk (28% vs. 5% of goat milk and 0% of others) than their counterparts. The most popular was bone/joint (12% of all products), followed by intestines (5%), heart (3%), muscle (3%) and brain (1%). 

**Images of animal/farm:** 55% of domestic and 34% of international products provided images of animal/farm on the pack. Compared with cow milks (31%), greater proportions of goat milks (81%) and other milks (90%) showed distinguishing images of animal/farm on their packs. 

**Images of product:** Milk images were found on 30% and 9% of domestic and international products, respectively. By milk source, product images were found on 33% of cow milks, 19% of goat milks and 10% of products made from other milk sources. 

#### 3.2.3. VWM from Third Parties

Certificates and patents were VWM from third parties on the pack of milks targeting COA. A greater proportion of domestic products indicated certificates than international products (63% vs. 38%, Table 7). Further, ‘imported’ and ‘recyclable’ certificates were observed most on international products. Production-related certificates, such as ISO 90001, Good Manufacturing Practice (GMP), Credit Management Systems (CMS) and Hazard Analysis and Critical Control Point (HACCP) appeared more commonly on domestic than international products. Certificates were more prevalent among goat milks (69%) than cow milks (52%), especially regarding production certificates, e.g., HACCP, ISO 90001, GMP, and CMS. Patents were found on only 5 domestic products (Table 7).

### 3.3. Compliance of Nutrition Claims with the Food Safety National Standards

**Figure 6:**
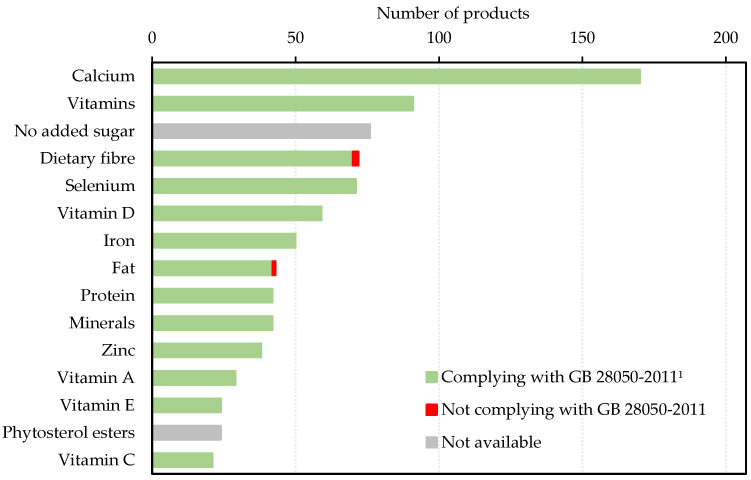
Number of milks targeting Chinese older adults made nutrition claims on specific nutrients that appeared on at least 5% of all products and the level of compliance. ^1^ GB 28050-2011 is the Food Safety National Standards for nutrition labelling of pre-packed foods issued by Ministry of Health of People’s Republic of China.

Amongst the nutrition claims made by milks targeting COA, three nutrition claims from three products failed to comply with Food Safety National Standards by either not meeting the requirements or not providing critical information (Figure 6 and Table 8). 

### 3.4. Alignment between Nutrition Claims and Nutrition Facts

**Figure 7:**
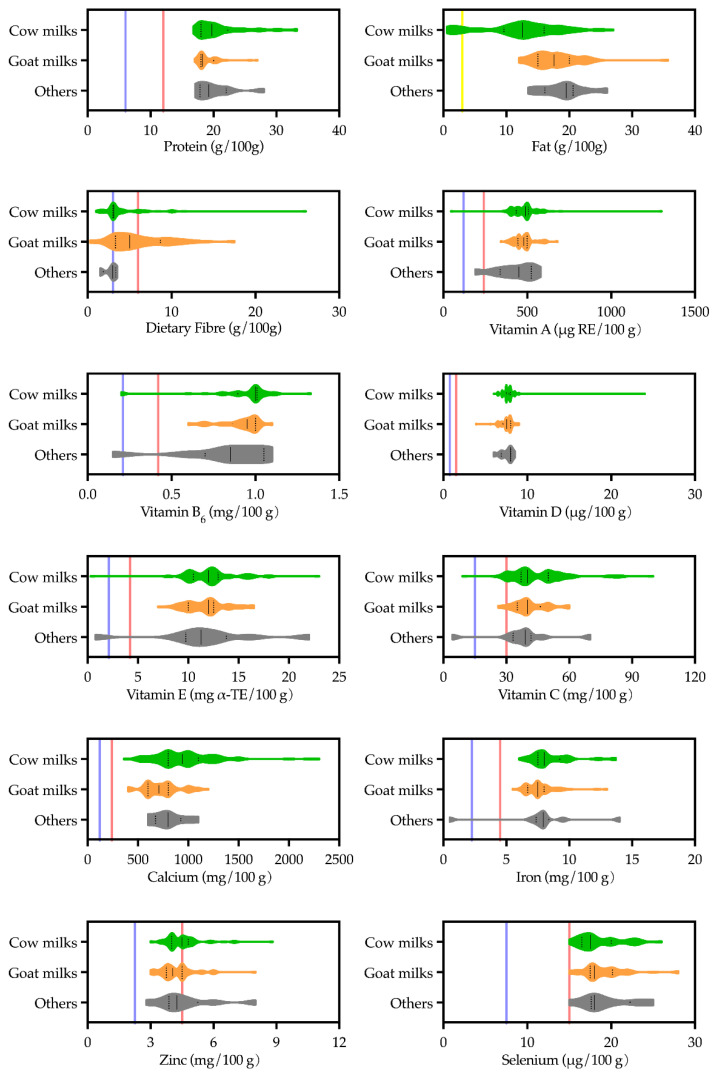
Nutrient concentrations for cow, goat and other milk powders targeting Chinese older adults. The solid line and dash line in the violin bars represent the median and quartiles, respectively. The colour lines are the minimum requirements to make nutrition content claims as high (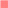), low (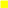), and contains (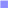), respectively, according to Food Safety National Standards for nutrition labelling of pre-packed foods (GB 28050-2011). ‘Others’ includes yak, camel and sheep milks.

Most nutrition claims were made in compliance with the regulation (Figure 6). However, the standard was made for all pre-packed foods, ‘one size fits all’. As milk powder, nutritional value was much greater than the standard criteria (Figure 7). For example, products that provided concentrations of protein, Vitamin D, Calcium and Selenium in their nutrition information tables were all eligible to claim ‘high’ for those nutrients (Figure 7). Additionally, goat milk products that provided Vitamin A, B_6_ and E concentrations in their nutrition information tables could all claim ‘high’ for those nutrients without violating the regulation (Figure 7). Therefore, to find out whether nutrition claims would help consumers choose amongst milks targeting COA, the alignment between nutrition claims and nutrition facts were further analysed.

**Figure 8:**
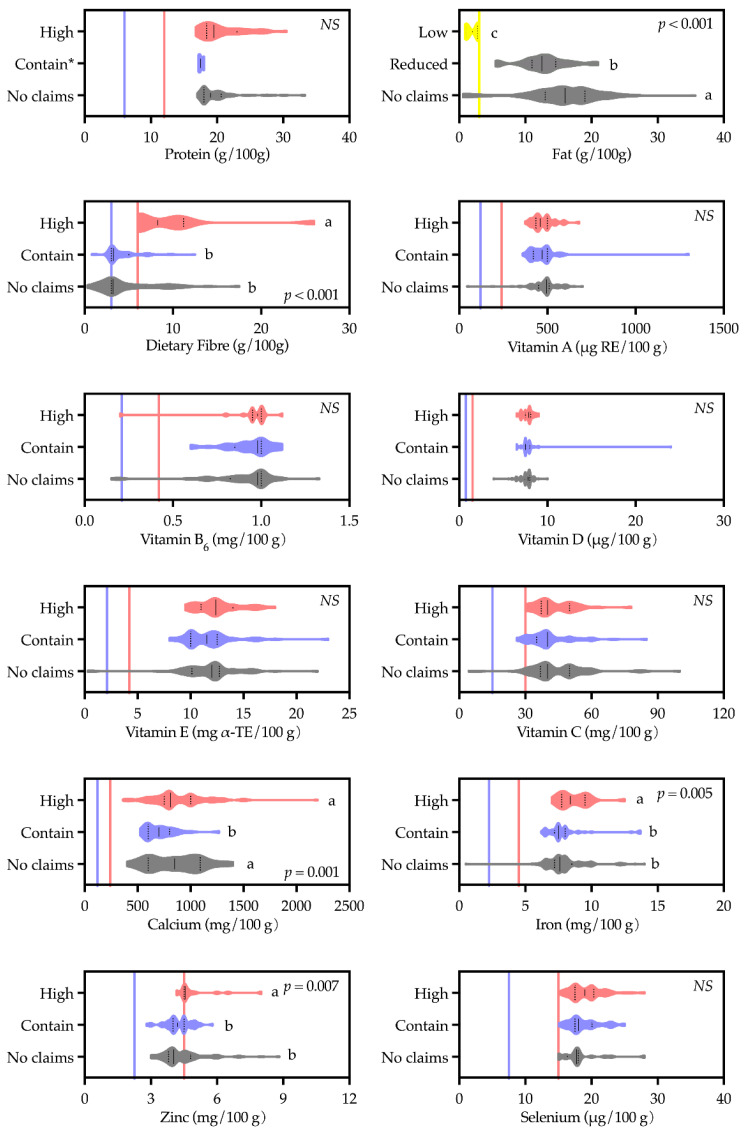
Nutrient concentrations for different levels of nutrition claim made by milk powders targeting Chinese older adults. The solid line and dash line in the violin bars represent the median and quartiles, respectively. The colour lines are the minimum requirements to make nutrition content claims as high (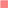), low (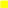), and contain (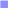), respectively, according to Food Safety National Standards for nutrition labelling of pre-packed foods (GB 28050-2011). *P*—significance level associated with Kruskal–Wallis *H* test. Bars with different subscripts were significantly different according to Dunn’s post hoc tests (*p* < 0.05), *NS*—not significant. * Mann–Whitney *U* test was conducted for comparing high protein and no claims on protein because the sample size of contain protein was too small.

Fat was the only nutrient where nutrition claims were aligned with concentration: milks claiming low in fat (*n* = 10, median = 2.0 g/100 g) and reduced fat (*n* = 33, median = 12.5 g/100 g) had significantly lower fat content than those did not make any fat claims (*n* = 161, median = 16.0 g/100 g). For the other nutrients, all ‘contain’ claims and most ‘high’ claims (except high in datary fibre, zinc and iron) did not reflect a significantly greater content of those nutrients compared to other products not making such claims (Figure 8).

## 4. Discussion

Milk targeting COA has a big market share in foods claiming suitability for older adults globally. This study explored this market by looking at milk targeting COA available both online and in physical stores in China and has provided a comprehensive survey of VWM appearing on the pack. Additionally, this study has made a unique contribution to categorising WM on food products by introducing an OTP VWM classification framework. This framework provides a foundation for consistent classification of OTP VWM, which can be applied to other food products with minor adjustments. Furthermore, attention has been given to nutrition-related WM by assessing compliance level of messaging with national regulations. Finally, the alignment between OTP nutrition claims and values presented in nutrition information tables was assessed. This section deliberates the key findings of this research, by discussing potential reasons behind each finding and how it sits within the broader relevant literature. It briefly lists the study’s strengths and limitations and how this line of research can come to further fruition in the future. 

### 4.1. Products

According to Mintel GNPD, instant foods from concentrates, such as milk powder, oatmeal, black sesame soup, etc., are the most popular food products designed for COA. In this study, a total of 207 unique milk targeting COA were identified, with 85% being produced by domestic brands and the rest 15% by international brands. Similar proportions were reported previously, where 87 milk powder products (85% with domestic brands and 15% with international brands) were identified in local Chinese markets in the year of 2017 and 2018 [49]. According to China Animal Husbandry and Veterinary Yearbook [50], 97.5% of China’s milk production was from cows in 2020 (33.4 million out of 35.3 million metric tons). However, this study found that the milks targeting COA were more diverse in terms of milk source. Milks sourced from five different ruminants were recorded; cow and goat were the main sources, but yak, camel and sheep milks represented 5% of the total. Notably, most goat milks were domestic products. 

### 4.2. Prevalence of OTP VWM on Milks Targeting COA

#### 4.2.1. Domestic vs. International

Domestic and international milks targeting COA used different VWM strategies. In particular, FOP nutrient profiling only appeared on international brand products. This might be explained by the fact that FOP nutrient profiling is more established and expected in countries that are the main exporters of milk to China, e.g., Oceania [51] and Europe [52]. Both nutrition and nutrient function claims were more frequent on products with domestic brand origins, partly since claims on international products with original packaging were not made in Chinese. With regards to claims on specific nutrients, calcium was, as expected, the most frequently mentioned nutrient regardless of brand origin. For other nutrients, however, domestic products more frequently claimed about vitamins in general, no added sugar, selenium, iron, zinc and phytosterol esters, whilst international brands focused on fat and specific vitamins (vitamin D, E, and C) more often. Domestic brands made considerable effort to highlight the abundance of minerals in their products, which appeared to be overlooked by international brands and might be explained by more in-depth local knowledge of the market segment’s unmet needs. Prior research, for instance, has continuously documented a significant deficiency of minerals amongst Chinese people [53,54,55,56]. 

Following a series of food safety scandals over the last two decades, the reputation of Chinese food products and Chinese consumer confidence in the domestic food industry were undermined fundamentally [57], especially for the dairy sector [58]. This appears to have shaped the way domestic products provide OTP VWM, which was in this study through increased VWM regarding ingredient (both textual and graphical), production, and certificates. Chinese manufacturers tended to provide information on farm details, production standard, traceability as well as the corresponding certificates, but such food safety related VWM was seldom mentioned on international products. Recently, it was reported that due to the heightened awareness of food safety in China, Chinese consumers expect to see food safety-related information not only on domestic, but also imported milk powders [59]. 

In terms of wellness-related VWM, similar proportions of domestic and international products provided textual VWM regarding physiological wellness. However, graphical schematic body parts, which were developed to mitigate potential barriers with textual messages, including going unnoticed, lack of interest and misunderstanding [60], were almost twice as prevalent amongst international brands, reflecting different strategies of communicating wellness benefits to their consumers. In addition to physical barriers, socio-psychological factors related to aging need to be taken into consideration when developing products targeted at the elderly [15]. Apparently, in terms of OTP VWM, a greater proportion of international products communicated to their consumers about psychological wellness, whilst sociological wellness, involving parent care from their offspring, was only found on domestic products (Table 5). This phenomenon is deeply rooted in the cultural belief of filial piety, which is believed to be the essential element holding together the Chinese familial system of care [61]. Filial piety is a Confucian concept that encompass a broad range of behaviours, including supplying food as daily maintenance, together with respect and sickness care [62]. It also explains why some domestic manufacturers use messages targeting the children and grandchildren of older adults rather than the end consumer themselves.

#### 4.2.2. Cow vs. Other Animals

Considering milk sources, similar proportions of cow and goat milk provided nutrition claims and nutrient functional claims, with more nutrients mentioned on the pack of goat milks. Further, cow milks provided more wellness-related VWM, both textually and graphically, than goat and other milks, whilst goat milks focused on production- and sensory-related VWM regarding mutton or goaty flavour removal, the major consumer-reported drawback of goat milk [63,64]. The overall prevalence of ingredient-related VWM (both textual and graphical) amongst different milk sources were similar, but cow milks focused on imported ingredients, whilst goat and other milks focused more on farm information. This finding is in line with previous studies found that milks produced by minor dairy species were distinct in terms of nutrition and production regions [65,66]. Additionally, third party certificates were more prevalent among goat and other milks, reflecting that one of the most important challenges for non-cow milks is to improve productivity and milking hygiene by standardised production processes [67]. 

### 4.3. Compliance and Alignment of Nutrition Claims

Governed by the relevant standardisation department under the Chinese government, there are more than 1000 National Standards on food safety covering many aspects [68]. For OTP WM of milks targeting COA, there were two relevant Food Safety National Standards made specifically for pre-packaged food labelling (GB 7718-2011, mandatory WM) and for pre-packaged food nutrition labelling (GB 28050-2011, voluntary WM). In this study, collectively, there were 769 verifiable nutrition claims made by the 207 milk products targeting COA, and only three claims (i.e., one “reduced fat” and two “contains dietary fibre”) from three products failed to comply with GB 28050-2011. Previously, Zhang reported 5.0% of fruit products available in Shanghai did not comply with the maximum residual limits for pesticides in food (GB 2763-2019) [69]. Wang summarised 540 pork hazard incidents based on 23,515 pork inspection cases from 2014 to 2016 (averaged at 2.3%), violating the hygienic standard for fresh (frozen) meat (GB 2707-2005), maximum levels of contaminants in foods (GB 2762-2012), general rules on pre-packaged food labelling (GB 7718-2011), etc. [70]. For claims on packaging to be effective, it is critical that they are understandable and, more importantly, trusted by the consumers [71]. In this study, by comparison, only 1.4% of milks targeting COA making nutrition claims failed the compliance test. It is worth mentioning that criteria of no added sugar and phytosterol esters were not available in GB 28050-2011 or any other Food Safety National Standards, making them less regulated. For example, a study in Taiwan found that 35 of 50 infant food products with “no added sugar” claims had high sugar content due to fruit ingredients [72]. 

Notably, GB 28050-2011 was a regulation made for *all* pre-packed food. A “One-size-fits-all” approach appears to have set the bar too low for milk powders (Figure 7), if differentiation amongst products of the same category is to be made. The study results showed that rather than indicating superior nutrition characteristics compared with competitors, most nutrition claims (both high and contains) highlighted the nutrition profile of milk powders in general compared with other categories of food. Nevertheless, research has found that nutrition claims on food product labels increased consumer perceptions of the presence of nutrients, perceived healthfulness of the product and thus intentions to consume the product [73]. Reading nutrition information tables might lead to reappraisals of a product’s healthfulness and potentially moderate the positive effects of nutrition claims on nutrition perceptions. The latest version of The Chinese Dietary Guidelines published in 2022 has specifically emphasised on the importance of understanding labels and information on pre-packed food as a consumer [74].

### 4.4. Strengths and Limitations

Two sources of product data were used to encompass both online and offline products. This is specifically relevant in China’s market where online shopping is rapidly claiming more market share, especially among imported food products [75,76]. This approach provided an advantage over similar works that only attend to one source of product information [77,78].

The fresh conceptualisation of VWM presented in this study, which is arguably more inclusive than prior attempts in the literature [79,80], is more capable of considering various sources, formats, and types of OTP information that directly or indirectly alludes to consumer well-being. Utilisation of a broad definition provided the opportunity to classify WM first through the state of being mandatory or voluntary, and then by origin, format, and content, accordingly (See Figure 1 and Table 1).

Whilst examining compliance of nutritional claims with local regulations seems to be the common situation in similar research [69,70], this study went above and beyond mere compliance with government regulations to examine meaningfulness of the claims when compared to other products in the same category. The results indeed suggested that GB 28050-2011, for instance by being set too low, failed to differentiate between milks with various claims on multiple occasions. Checking the alignment between the nutrition information table and nutritional claims, hence, painted a more accurate picture here, one that should be helpful to the consumer and perhaps policymakers. This study relied on data provided in the nutritional information table as an indicator of composition. Future research could improve on this by undertaking independent laboratory nutritional analysis of the products 

## 5. Conclusions

Based on 207 milk products targeting COA identified from both online and offline retailers, products with different brand origins (i.e., domestic vs. international) and milk sources (i.e., cow vs. goat vs. other) had different strategies of providing OTP VWM. Specifically, domestic products more commonly made nutrition claims on minerals, vitamins in general and no added sugar, whilst international products claimed fat and individual vitamins more often. When communicating wellness-related well-being, domestic products focused on providing textual physiological and sociological VWM, but international products had more graphical physiological and psychological VWM. Domestic brands tended to emphasise production standard, traceability, milk source details as well as the corresponding certificates, but such food safety related VWM was seldom mentioned by international products. Goat and other milks differentiated themselves by providing more VWM regarding ingredient (farm information), production, sensory and third-party certificates than cow milks. When making nutrition claims, only three products failed to comply with the government regulations by either not specifying the nutrient concentration or the stated concentration being lower than the criteria. However, except for fat (both claims of low and reduced), all “contains” claims and most “high” claims on the other nutrients did not guarantee a significantly greater content of those nutrients, compared to other milks not making such claims. This phenomenon was mainly due to the ineffectiveness of current regulations which have meant most OTP nutrition claims, rather than indicate superior nutrition characteristics of a product compared to its competitors, highlight the nutrition profile of milk powders in general, compared to other categories of food. The findings created a comprehensive picture of OTP VWM for milks targeting older adults in China, providing useful information for consumer, domestic, and international dairy industries, and policymakers. Future work should be carried out to understand consumers perceptions of specific VWM and their relative impacts on milk choice behaviour.

## Figures and Tables

**Table 1:** Detailed classifications and working definitions of on-the-pack textual voluntary well-being messaging on milks targeting Chinese Older Adults.

Classification	Detail	NRVs	Illustrative Examples
Nutrition	Nutrition claim	NRVs	high in protein; contain selenium
	Nutrient function claim	a description or declaration of the role of a nutrient in growth, development and physiological function	calcium helps the growth of bone and tooth
	Front-of-pack nutrient profiling	the standardised icons on the front of pack briefly showing nutrition information	energy: 391 kJ, 5% of NRVs ^1^
Ingredient	Imported ingredients	a statement showing that the milk source or other ingredients were imported from outside mainland China	imported probiotics; New Zealand imported milk source
	Farm information	a statement describing the farm location, environment, feed quality, animal breed, raw milk quality and whether operated by the manufacturer	farm location: Fuping, the land of goat; Holstein cow; premium milk source; self-operated farm
	Other specified ingredients	Other specified nondigestible supplements, food additives and mixtures of nutrients	prebiotics; probiotics; fish oil; herb extracts
Brand	Slogan	a catchphrase representing a product and a company to convey the values of the brand	better nutrition, healthier life; high quality & classical
	Profile	a statement showing the history, reputation and strength of the company or/and the brand and what the public think and feel about the manufacturer	since 1953; cooperating with universities for professional formulas
Wellness	Physiological	a statement specifying the physical challenges for COA and the health benefits brought by the product	bone density decreases as aging; ‘healthy bone’ formula with rich milk minerals
	Psychological	a statement encouraging COA to be optimistic and to stay physically fit and mentally positive	it’s a fresh start, everything is achievable if you have a positive mindset and are physically active
	Sociological	a statement showing family connections potentially brought about by the product	come home and visit your parents
Production	Quality control	a process by which entities review the quality of all factors involved in production	strict quality control
	Traceability	the capability to trace all processes from raw materials to products	traceable and reliable
	Mutton flavour removal	the techniques to removal mutton flavour from goat milks	vacuum evaporation is used to remove mutton flavour
	Other specified methods	other processing or production techniques and methods specified	nitrogen food packaging; wet-mix process
Sensory	Flavour	a description of taste, smell or their combination	classic milky flavour; rich & fragrant
	Texture	a description of tactile characteristics and mouthfeel	smooth mouthfeel
	Appearance	a description of the particle sizes and colour	fine, loose, and milky white

^1^ NRVs represents Nutrient Reference Values.

**Table 2:** Category, classification and criteria of nutrition claims according to GB 28050-2011 ^1^.

Category	Classification	Synonym and Alternative	Criteria
Content claim	High	rich, good source of	≥30% NRVs ^2^/100 g
	Low	less	≥15% NRVs/100 g
	Skim ^3^	-	≤1.5 g/100 g
	Contain	source of, added, provide, include, have, enhanced, NOTHING	≥15% NRVs/100 g
	Not contain	without, zero, no, 0%, not include	≤0.5 g/100 g
Comparison claim	Increased	XX% more	25% more ^4^
	Reduced	XX% less or decreased	25% less ^4^

^1^ GB 28050-2011 is the Food Safety National Standards for nutrition labelling of pre-packed foods issued by Ministry of Health of People’s Republic of China. ^2^ NRVs represents Nutrient Reference Values. ^3^ For fat content only. ^4^ Compared to the reference food, which is of the same type and well-known by consumers.

**Table 3:** Summary of eligible products.

		Number of Products	Percentage
Source ^1^	JD.com	195	94%
	Mintel GNPD ^2^	77	37%
Milk Type	Powder	204	99%
	Liquid	3	1%
Brand origin	Domestic	175	85%
	International	32	15%
Animal	Cow	123	59%
	Goat	74	36%
	Yak	4	2%
	Camel	3	1.5%
	Sheep	3	1.5%

^1^ 65 products from both sources. ^2^ Mintel’s global new product database.

**Table 4:** Percentage and number of milks targeting Chinese older adults providing textual voluntary well-being messaging by total, brand origin and milk source ^1^.

	Total (*n* = 207)	Brand Origin		Milk Source		
		Domestic (*n* = 175)	International (*n* = 32)	Cow (*n* = 123)	Goat (*n* = 74)	Others (*n* = 10)
Nutrition	95% (197)	98% (172)	78% (25)	95% (117)	97% (72)	80% (8)
Nutrition claim	95% (196)	98% (171)	78% (25)	95% (117)	97% (72)	70% (7)
Nutrient function claim	58% (120)	61% (107)	41% (13)	58% (71)	59% (44)	50% (5)
FOP ^2^ nutrient profiling	4% (8)	0% (0)	23% (8)	7% (8)	0% (0)	0% (0)
Ingredient	74% (154)	73% (127)	84% (27)	73% (90)	78% (58)	60% (6)
Imported ingredients	25% (51)	15% (26)	78% (25)	34% (42)	11% (8)	10% (1)
Farm information	34% (70)	39% (69)	3% (1)	27% (33)	43% (32)	50% (5)
Other specified ingredients	41% (84)	42% (74)	31% (10)	38% (47)	47% (35)	20% (2)
Brand	51% (106)	51% (90)	50% (16)	57% (70)	41% (30)	60% (6)
Slogan	44% (91)	44% (77)	44% (14)	46% (56)	39% (29)	60% (6)
Profile	15% (31)	17% (29)	6% (2)	18% (22)	11% (8)	10% (1)
Wellness	32% (67)	32% (56)	34% (11)	42% (52)	19% (14)	10% (1)
Physiological	23% (48)	23% (40)	25% (8)	30% (37)	14% (10)	10% (1)
Psychological	10% (21)	7% (13)	25% (8)	15% (19)	3% (2)	0% (0)
Sociological	6% (12)	7% (12)	0% (0)	7% (8)	5% (4)	0% (0)
Production	26% (67)	31% (56)	0% (0)	25% (52)	31% (14)	0% (0)
Quality control	9% (19)	11% (19)	0% (0)	11% (14)	7% (5)	0% (0)
Traceable	3% (7)	4% (7)	0% (0)	2% (3)	5% (4)	0% (0)
Mutton flavour removal	5% (10)	6% (10)	0% (0)	0% (0)	14% (10)	0% (0)
Other specified methods	14% (30)	17% (30)	0% (0)	16% (20)	14% (10)	0% (0)
Sensory	15% (31)	15% (27)	13% (4)	13% (16)	20% (15)	0% (0)
Flavour	14% (28)	14% (24)	13% (4)	11% (14)	19% (14)	0% (0)
Texture	6% (12)	7% (12)	0% (0)	7% (8)	5% (4)	0% (0)
Appearance	2% (4)	2% (3)	3% (1)	1% (1)	4% (3)	0% (0)

^1^ Percentages in a column were calculated based on the number of products given in the headings. ^2^ FOP: front of pack.

**Table 5:** Verbatim of all instances of sociological wellness voluntary well-being messaging (VWM) about family.

Brand Origin	Milk Source	Sociological Wellness VWM
Domestic	Cow/goat	“Come home and visit your parents.”
Domestic	Cow	“Ensure the health of your parents.”
Domestic	Cow	“Take care of your parents like the kids.”
Domestic	Cow	“Care is the best gift.”
Domestic	Cow/goat	“Understanding what your parents need.”
Domestic	Goat	“Love your parents back.”
Domestic	Cow	“Thank you, mum and dad.”

**Table 6:** Percentage and number of milks targeting Chinese older adults providing graphical voluntary well-being messaginging by total, brand origin and milk source ^1^.

	Total (*n* = 207)	Brand Origin		Milk Source		
		Domestic (*n* = 175)	International (*n* = 32)	Cow (*n* = 123)	Goat (*n* = 74)	Others (*n* = 10)
Older adults	52% (107)	53% (93)	44% (14)	59% (72)	43% (32)	30% (3)
Form:						
Photograph	31% (65)	31% (54)	34% (11)	44% (54)	14% (10)	10% (1)
Cartoon	20% (42)	22% (39)	9% (3)	15% (18)	30% (22)	20% (2)
Number of people:						
One	8% (17)	9% (15)	6% (2)	9% (11)	7% (5)	10% (1)
Two	43% (88)	43% (76)	38% (12)	48% (59)	36% (27)	20% (2)
Gender:						
Male	7% (14)	8% (14)	0% (0)	7% (8)	8% (6)	0% (0)
Female	3% (6)	2% (4)	6% (2)	3% (4)	1% (1)	10% (1)
Both	42% (86)	43% (75)	34% (11)	48% (59)	34% (25)	20% (2)
Ethnic group:						
Southeast Asian	33% (69)	37% (65)	13% (4)	39% (48)	24% (18)	30% (3)
European	9% (19)	6% (11)	25% (8)	12% (15)	5% (4)	0% (0)
Schematic	18% (38)	16% (28)	31% (10)	28% (34)	5% (4)	0% (0)
Bone/joint	12% (25)	11% (20)	16% (10)	17% (21)	5% (4)	0% (0)
Intestines	5% (11)	3% (6)	16% (5)	9% (11)	0% (0)	0% (0)
Heart	3% (7)	3% (5)	6% (2)	5% (6)	1% (1)	0% (0)
Muscle	3% (6)	3% (5)	3% (1)	3% (4)	3% (2)	0% (0)
Brain	1% (3)	2% (3)	0% (0)	2% (3)	0% (0)	0% (0)
Animal/farm	52% (107)	55% (96)	34% (11)	31% (38)	81% (60)	90% (9)
Product	27% (56)	30% (53)	9% (3)	33% (41)	19% (14)	10% (1)

^1^ Percentages in a column were calculated based on the number of products given in the headings.

**Table 7:** Percentage and number of milks targeting Chinese older adults providing third party originated voluntary well-being messaging, certificates and patents, by total, brand origin and by milk source ^1^.

	Total (*n* = 207)	Brand Origin		Milk Source		
		Domestic (*n* = 175)	International (*n* = 32)	Cow (*n* = 123)	Goat (*n* = 74)	Others (*n* = 10)
Certificate	59% (123)	63% (111)	38% (12)	52% (64)	69% (51)	80% (8)
HACCP	32% (66)	37% (65)	3% (1)	24% (30)	47% (35)	10% (1)
ISO 90001	31% (64)	36% (63)	3% (1)	24% (29)	46% (34)	10% (1)
GMP	18% (38)	21% (37)	3% (1)	10% (12)	34% (25)	10% (1)
Recyclable	14% (29)	13% (22)	22% (7)	11% (14)	15% (11)	40% (4)
Halal	10% (20)	11% (19)	3% (1)	11% (14)	4% (3)	30% (3)
CMS	9% (19)	11% (19)	0% (0)	2% (3)	20% (15)	10% (1)
ISO 14001	8% (17)	10% (17)	0% (0)	13% (16)	1% (1)	0% (0)
QS	7% (15)	9% (15)	0% (0)	8% (10)	5% (4)	10% (1)
Imported certificates	5% (10)	0% (0)	31% (10)	7% (9)	1% (1)	0% (0)
Patent	2% (5)	3% (5)	0% (0)	2% (3)	3% (2)	0% (0)

^1^ Percentages in a column were calculated based on the number of products given in the headings. Abbreviations: HACCP—Hazard Analysis and Critical Control Point; ISO—International Organization for Standardization; GMP—Good Manufacturing Practice; CMS—Credit Management Systems; QS—Quality Standard.

**Table 8:** Products failing to comply with GB 28050-2011 ^1^.

Brand Origin	Milk Source	Nutrition Claim	Nutrition Fact ^2^	Criteria
Domestic	Cow	Comparison claim: reduced fat	Not specified	≥25%
Domestic	Cow	Content claim: contains dietary fibre	1.25 g/100 g	≥3 g/100 g
Domestic	Goat	Content claim: contains dietary fibre	0.8 g/100 g	≥3 g/100 g

^1^ GB 28050-2011 is the Food Safety National Standards for nutrition labelling of pre-packed foods issued by Ministry of Health of People’s Republic of China. ^2^ Nutrition facts were sourced from on-the-pack nutrition information table and statements.

## Data Availability

Data is contained within the article.
